# PharmFreq: a comprehensive atlas of ethnogeographic allelic variation in clinically important pharmacogenes

**DOI:** 10.1093/nar/gkae1016

**Published:** 2024-11-14

**Authors:** Roman Tremmel, Yitian Zhou, Mahamadou D Camara, Sofiene Laarif, Erik Eliasson, Volker M Lauschke

**Affiliations:** Dr Margarete Fischer-Bosch Institute of Clinical Pharmacology, 70376 Stuttgart, Germany; University of Tuebingen, 72074 Tuebingen, Germany; Department of Physiology and Pharmacology, Karolinska Institutet and Center for Molecular Medicine, Karolinska Institutet and University Hospital, 17165 Stockholm, Sweden; Department of Physiology and Pharmacology, Karolinska Institutet and Center for Molecular Medicine, Karolinska Institutet and University Hospital, 17165 Stockholm, Sweden; Department of Laboratory Medicine, Karolinska Institutet, 14152 Stockholm, Sweden; Department of Laboratory Medicine, Karolinska Institutet, 14152 Stockholm, Sweden; Dr Margarete Fischer-Bosch Institute of Clinical Pharmacology, 70376 Stuttgart, Germany; University of Tuebingen, 72074 Tuebingen, Germany; Department of Physiology and Pharmacology, Karolinska Institutet and Center for Molecular Medicine, Karolinska Institutet and University Hospital, 17165 Stockholm, Sweden; Department of Pharmacy, the Second Xiangya Hospital, Central South University, 410013 Changsha, China

## Abstract

Genetic polymorphisms in drug metabolizing enzymes, drug transporters as well as in genes encoding the human major histocompatibility complex contribute to inter-individual differences in drug efficacy and safety. The extent, pattern and complexity of such pharmacogenetic variation differ drastically across human populations. Here, we present PharmFreq, a global repository of pharmacogenetic frequency information that aggregates frequency data of 658 allelic variants from over 10 million individuals collected from >1200 studies across 144 countries. Most investigations were conducted in East Asian and European populations, accounting for 29.4 and 26.6% of all studies, respectively. We find that the number of studies per country and aggregated cohort size correlated significantly with population size (R = 0.55, *P*= 3*10^−9^) and country gross domestic product (R = 0.43, *P*= 2*10^−6^) with overall population coverage varying between 5% in Estonia to < 0.001% in many countries in Sub-Saharan Africa and Asia. All frequency data are openly accessible via a web-based interactive dashboard at pharmfreq.com that facilitates the exploration, visualization and analysis of country- and population-specific data and their inferred phenotypic consequences. PharmFreq thus presents a comprehensive, freely available resource for pharmacogenetic variant frequencies that can inform about ethnogeographic pharmacogenomic diversity and reveal important inequities that help to focus future research efforts into underrepresented populations.

## Graphical Abstract

**Graphical Abstract:**
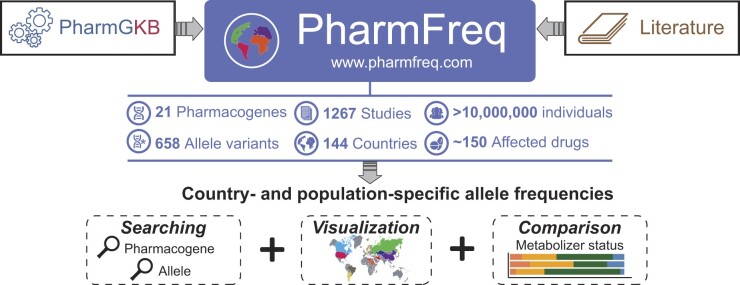

## Introduction

The response to pharmacological treatment is majorly impacted by genetic variation. Specifically, polymorphisms in drug metabolizing enzymes, drug transporters and genes encoding the human major histocompatibility complex (MHC) are associated with inter-individual differences in efficacy and safety of more than 150 medicines (1–3). On average, each patient harbors genetic variations that affect the response to at least 10 medications, demonstrating that actionable pharmacogenetic variants combined are highly prevalent (4). The extent of pharmacogenetic testing of individual patients is increasing in recent years, but overall coverage still remains low (5). In the absence of such personal genetic information, frequency data at the population level can already offer powerful resources to guide rational prescribing and precision public health.

The extent, pattern and complexity of pharmacogenetic variation differs drastically between human populations (6). One striking example is the genetic variability in *CYP2D6*, which encodes a metabolic enzyme that metabolizes around 20% of all clinically used drugs (7). Overall, 40–80% of all individuals harbor at least one *CYP2D6* allele with altered function; however, while CYP2D6*10, a haplotype characterized by the two variants g.5119C > T (p.P34S) and g.9200G > C (p.S486T), is the major cause of reduced CYP2D6 activity in East Asian populations, CYP2D6*17, which comprises three missense variants, g.6041C > T (p.T107I), g.7870C > T (p.R296C) and g.9200G > C (p.S486T), is most common in African populations and the splicing defect CYP2D6*4 (g.6866G > A) is most frequent in Europeans (8–10).

While these and other ethnogeographic differences are well established, there are only few resources available that consolidate the available data and allow to easily obtain an overview of the global distribution of these important pharmacogenetic alleles. The Human Gene Mutation Database (HGMD), dbSNP and the Frequency of INherited Disorders database (FINDbase) provide the possibility to obtain global frequency information of pharmacogenetic variants using a user-friendly interface (11,12). However, no haplotype data is available, which does hence not allow for direct genotype-to-phenotype translation. Other resources based on population-scale sequencing projects, such as gnomAD (13), only yield highly aggregated frequencies at the population level and do not offer high-resolution information about variations in different countries of ethnogeographic groups. Similarly, while key pharmacogenomic databases, such as PharmGKB (14), serve as powerful repositories of pharmacogenetic allele frequencies, this resource does not provide an integrated and easily accessible overview of global variability patterns.

PharmFreq provides the first collated repository and search tool for worldwide high-resolution pharmacogene allele frequency data. Overall, the database integrates frequency information of 658 alleles from more than 10 million individuals across 144 countries. These data were extracted from over 1200 studies using systematic mining coupled with careful expert curation and collectively affect safety and efficacy of > 150 medicines. The results are provided in an intuitive and versatile graphical user interface (GUI), allowing users to explore the ethnogeographic variability of genetically encoded drug response differences and identify outlier populations that might benefit from therapeutic adjustments. Furthermore, application to highly admixed and heterogeneous populations allows for direct comparisons of available genetic information with the real-world national demographics. These data thereby quantify the underrepresentation of different ethnic groups in pharmacogenomic research and provide much needed objective information to overcome pharmacogenomic inequities.

## Materials and methods

### Frequency data collection and curation

Pharmacogenes were selected based on their clinical actionability as defined by the Clinical Pharmacogenetics Implementation Consortium (CPIC). Specifically, all gene-drug pairs with CPIC level A were included with the exception of *CFTR*, since the pharmacogenetic guidance for this gene is specific to cystic fibrosis patients, not for the general population. This approach resulted in the selection of a total of 21 pharmacogenes and 658 alleles, pertaining to a total of 150 gene-drug pairs. Frequency data were collected from PharmGKB and PubMed following a systematic literature search with the following search terms [gene name] AND (genotype OR allele OR frequency OR minor allele OR variant OR population OR ethnic OR ethnicity OR [country]). Only original publications in English were included. In alignment with PharmGKB filter criteria, studies were excluded if information about the geographic origin of the population was lacking, allele or genotype frequencies were not conclusively reported or the method by which the genes were genotyped was not clearly indicated. In addition, frequency information from gnomAD, AllOfUs, allelefrequencies.net, the Estonian Biobank and the UKBioBank were included, resulting in the inclusion of a total of 1267 studies comprising genetic data from >10 million individuals (Table 1; Supplementary Table S1). Identified studies were reviewed and detailed meta-information about allele frequency, cohort size, and ethnical background (e.g. population, ancestry) of the samples was collated. Data collection included all studies available by 1 June 2024. Frequency information was mapped to biogeographic groups using the grouping system established for pharmacogenetic research (15) as well as to specific countries using the corresponding alpha-2 code (ISO 3166–1) where possible. Only alleles that have a frequency >0.001% in at least one country or population are shown. Star allele definitions and functional annotations were obtained from PharmVar (16,17).

**Table 1.** General metrics of data included in PharmFreq. Note that the number of studies listed exceeds the number of studies included in PharmFreq as some studies cover multiple regions

Geographical region	Aggregated cohort size	Number of studies
Central and South Asian	191227	128
East Asian	526227	502
European	2681961	467
Middle Eastern & North African	235898	147
North American	5856097	176
Oceanian	19161	27
South American	1840465	107
Sub-Saharan African	155952	162

### Database construction

Based on the structure of the different data sources, we used R and the fst package (18) to construct lightweight data frames for frequency, haplotype and functional data. Database queries, and mutating joins were written using dplyr (19), purrr (20) and tidyr (21). The database and interactive dashboard were hosted using Rshiny (22) in a dockerized container at a cloud-based app hosting service. Communication between the browser and server uses the SSL/TLS protocol for encryption and authentication (HTTPS). The leaflet package (23) was applied to visualize the frequency data using choropleth maps. Curated raw and aggregated data from PharmFreq are freely available through download functions at all data layers to facilitate further custom analyses or visualization using end-user tools. PharmFreq is fully accessible and legible on mobile phones and tablet screens. Of note, users can contribute frequency data of additional cohorts via submission to the PharmFreq working group via the Contact function.

### Statistical methods

The user can select between display of allele frequencies across countries or geographical groups aggregated using the median or a weighted median approach implemented in matrixStats (24) using the cohort sizes as weighting factor using the R package. For regions with more than two studies, also confidence intervals or the absolute deviations from the median are provided. Country population estimates were obtained from World Population Prospects: The 2019 Revision (http://population.un.org/wpp). National gross domestic products (GDPs) per capita were retrieved from the World Bank's application programming interaction with the ‘NY.GDP.PCAP.KD’ indicator.

## Results

### Data statistics and content

**Figure 1.**
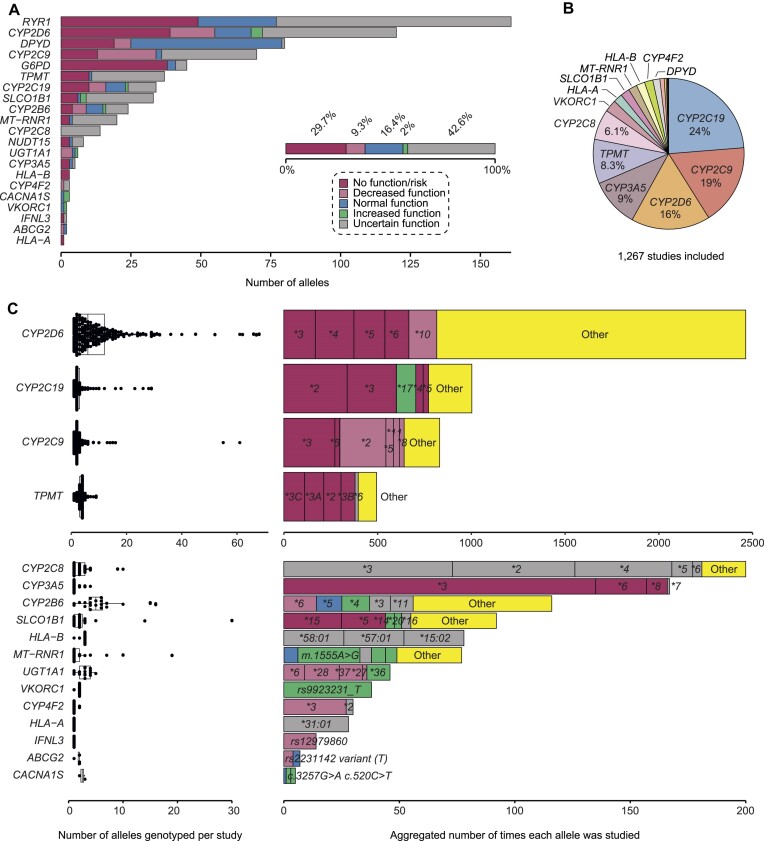
Overview of pharmacogenes and alleles in PharmFreq. (**A**) Stacked bar plot showing the number of included alleles for each pharmacogene. The functional consequences are indicated using the different colors. (**B**) The total number of studies distributed over all studied pharmacogenes. (**C**) Distribution of the number of alleles genotyped per study (left) and a cumulative overview of the number of times the different alleles were studied for each pharmacogene (right).

PharmFreq contains worldwide allele frequency data of 21 clinically important pharmacogenes, including genes encoding drug metabolizing enzymes, drug transporters, HLA genes and other genes with existing drug and dosage guideline (Table 2). In total, PharmFreq currently includes genotyping data from > 10 million individuals, providing population-scale coverage for 658 alleles distributed across 144 countries. The largest numbers of alleles are covered for *RYR1, DPYD, CYP2D6* and *CYP2C9* (Figure 1A). Almost half of the alleles are associated with functional consequences, mostly resulting in loss-of-function or increased risk (29.7%) or decreased function (9.3%). In contrast, the fraction of increased function alleles is considerably lower (2%; Figure 1A, inlet). In the current release, data from 1267 studies and databases are considered with the largest numbers reporting allele frequencies of the CYP2 family members *CYP2C19, CYP2C9* and *CYP2D6* (Figure 1B). In contrast, coverage is substantially lower for *DPYD* and *TPMT*, despite their established clinical importance. The vast majority of studies tested < 10 candidate variants, whereas <10 investigations interrogated more than 40 variants in a single study (Figure 1C). For most genes, investigations were skewed towards individual alleles. This is most pronounced for *CYP3A5, HLA-A, IFNL3* and *CYP4F2* where >80% of the available data refers to a single allele.

**Table 2.** Selected dosing guideline pertaining to pharmacogenes in PharmFreq

		Drugs with recommendation^a^		
Gene	Number of alleles covered	CPIC	DPWG	Other
***CYP2B6***	23	Efavirenz, sertraline	Efavirenz	
***CYP2C19***	33	Amitriptyline, citalopram, clomipramine, clopidogrel, dexlansoprazole, doxepin, escitalopram, imipramine, lansoprazole, omeprazole, pantoprazole, sertraline, trimipramine, voriconazole	Citalopram, clomipramine, clopidogrel, escitalopram, imipramine, lansoprazole, omeprazole, pantoprazole, sertraline, voriconazole	Antidepressants, clopidogrel, voriconazole
***CYP2C9***	69	Celecoxib, flurbiprofen, fluvastatin, fosphenytoin, ibuprofen, lornoxicam, meloxicam, phenytoin, piroxicam, tenoxicam, warfarin	Phenytoin, siponimod, warfarin	Acenocoumarol, fluindione, warfarin
***CYP2D6***	119	Amitriptyline, atomoxetine, clomipramine, codeine, desipramine, doxepin, fluvoxamine, hydrocodone, imipramine, nortriptyline, ondansetron, paroxetine, tamoxifen, tramadol, trimipramine, tropisetron	Amitriptyline, aripiprazole, atomoxetine, brexpiprazole, clomipramine, codeine, doxepin, eliglustat, flecainide, haloperidol, imipramine, metoprolol, nortriptyline, paroxetine, pimozide, propafenone, risperidone, tamoxifen, tramadol, venlafaxine, zuclopenthixol	Antidepressants, codeine, tamoxifen
***CYP3A5***	4	Tacrolimus	Tacrolimus	Tacrolimus
***CYP4F2***	2	Warfarin		
***DPYD***	78	Capecitabine, fluorouracil	Capecitabine, flucytosine, fluorouracil, tegafur	Capecitabine, fluorouracil, tegafur
***HLA-A***	1	Carbamazepine	Carbamazepine	Carbamazepine
***HLA-B***	3	Abacavir, allopurinol, carbamazepine, fosphenytoin, oxcarbazepine, phenytoin	Abacavir, allopurinol, flucloxacillin, lamotrigine, oxcarbazepine	Allopurinol, carbamazepine
***NUDT15***	7	Azathioprine, mercaptopurine, thioguanine	Azathioprine, mercaptopurine, thioguanine	
***SLCO1B1***	32	Atorvastatin, fluvastatin, lovastatin, pitavastatin, pravastatin, rosuvastatin, simvastatin	Atorvastatin, simvastatin	Statins
***TPMT***	36	Azathioprine, mercaptopurine, thioguanine	Azathioprine, mercaptopurine, thioguanine	Azathioprine, cisplatin, mercaptopurine
***UGT1A1***	5	Atazanavir	Irinotecan	Irinotecan
***VKORC1***	1	Warfarin	Warfarin, acenocoumarol, phenprocoumon	Acenocoumarol, fluindione, warfarin

^a^Testing info, dosing info or alternative drug recommendation.

### User interface and features

**Figure 2.**
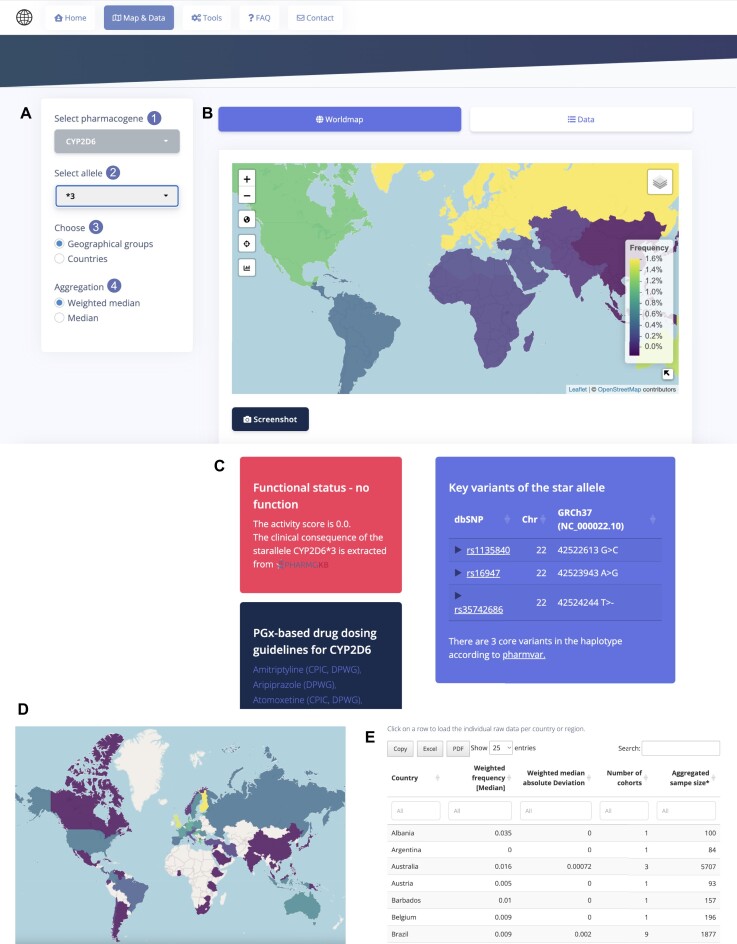
Interface for allele frequencies illustration. (**A**) Drop-down menus for selecting the specific pharmacogene (1) and allele (2) of interest, which should be displayed on the world map. Frequencies can be displayed per aggregated geographical group or per country (3), and different aggregation methods of the frequency data can be selected (4). (**B**) An exemplary world map illustrating frequencies of the selected allele across geographical groups is shown using CYP2D6*3 as an example. Frequencies are color-coded automatically using a dynamic look-up table (LUT) from zero to the highest frequency. The map is interactive and clicking on a country triggers a pop-up window showing all available studies for the given selection. Furthermore, the map can be screenshot for further use. (**C**) Important additional data, including the constituting variations for the selected allele, its functional status and clinically used drugs with associated actionable guidelines are shown below the map. (**D**) Corresponding data with resolution at the country level. (**E**) The data tab allows easy access to the aggregated numerical frequencies of all countries and provides a free download functionality for all underlying data.

The PharmFreq landing page introduces the basic statistics of the presented data, including the number of studies and analyzed pharmacogenes/alleles included in its most recent release. With a single click via the ‘Let's start’ button, the user is directed to the Map&Data dashboard, in which all population frequency data can be easily displayed and accessed. The user has the possibility to select a pharmacogene and a corresponding allele from the respective drop-down menus on the left side (Figure 2A; inlets 1 and 2). Selection triggers the display of a world choropleth map in the main panel of the website together with detailed information about the allele (Figure 2B). The functional consequences of the allele are shown as defined by PharmGKB together with associated drugs that contain pharmacogenetic dosing guidelines for this gene by pharmacogenetic expert work groups (CPIC and DPWG; Figure 2C). Furthermore, the defining variants for the selected allele based on the latest PharmVar definitions are shown with hyperlinks that allow the user to directly access the corresponding entry in the dbSNP resource. This approach provides users with a clear understanding of the constituting genetic architecture of the selected allele.

Users can select the preferred geographic resolution and can choose the preferred method for aggregation of frequency data (unweighted or weighted by the cohort size; Figure 2A; inlets 3 and 4). Depending on the selection, allele frequencies are then mapped by geographical group (Figure 2B) or country (Figure 2D) using a spectral divergent color scheme. By hovering the cursor over a country or geographical group with available data, information about the allele frequency, the number of studies as well as the aggregated sample size is shown. Single-clicking on a country displays a modal dialog in the client browser with the underlying country-specific data. The aggregated data of all countries can also be accessed in a tabular form using the data tab in the main panel (Figure 2E). In each rendered table, buttons for free download of the respective data as tab-delimited text file are available below the table to enable further analysis or visualization. To further facilitate the analysis of population frequency data, we implemented a toolbox in addition to the Map&Data dashboard to illustrate frequency differences across genes and populations.

### Pharmacogenetic analysis toolkit

**Figure 3.**
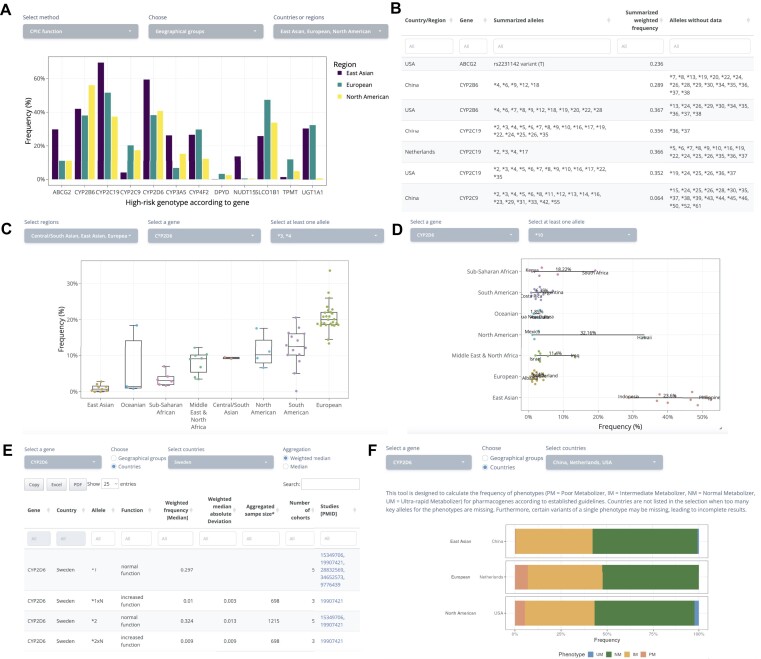
PharmFreq toolkit for analyzing population-scale frequency data. (**A**) Using the ‘High-risk genotype frequency tool’, the aggregated frequency of risk alleles can be compared across different geographic groups or countries. (**B**) Below the interactive column plot, a table is shown that presents all alleles included in the frequency analysis. Alleles for which there are no data are shown to pinpoint important gaps in current knowledge. (**C**) Using the ‘Frequency comparison tool’, the frequencies of alleles of interest can be compared across biogeographic groups of interest. (**D**) Using the ‘Intra-biogeographic variability tool’, frequency differences within biogeographic groups can be displayed and countries with highest and lowest frequencies are labeled. This provides an overview of the heterogeneity within major, often aggregated populations. (**E**) The ‘Country data tool’ uses one or more countries/geographical groups and genes as input to conveniently provide all available data for the given selection in a tabulated, downloadable form. (**F**) Upon selection of a gene, the ‘Metabolizer status tool’ can calculate the frequencies of UMs, normal metabolizers (NMs), intermediate metabolizers (IMs) and poor metabolizers (PMs) for one or more countries/geographic groups using the established CPIC algorithms for phenotype inference. Metabolizer distributions are shown both as stacked bar plots and numerical tables.

Individuals carrying alleles that decrease or increase enzyme functions may show diverse drug metabolizer phenotypes and, consequently, can be at risk of low drug efficacy or adverse drug reactions. By leveraging large-scale functional allele frequency data from PharmFreq, the fraction of individuals carrying risk alleles can be directly estimated on the basis of all consolidated data using the ‘High-risk genotype frequency’ tool. Taking the selection of East Asia, Europe and North America as examples, East Asia harbors the highest number of individuals expected to carry risk alleles in *CYP2D6* and *NUDT15*, whereas *CYP2C9* and *DPYD* risk variants are most common in Europe and North America (Figure 3A). When switching to the method from Dunnenberger et al. (25) that only considers selected variants in 12 commonly tested genes, results are overall similar; however, selection of country-resolution data reveals major differences in *VKORC1* risk allele frequencies and *CYP3A5* expressor status are identified between China and the US, indicating high variability in warfarin and tacrolimus dose requirements (Figure 3B). The alleles included in the evaluation for each gene and country as well as the alleles for which data is missing are summarized in the table below.

In addition, frequency of one single allele or aggregated frequency of multiple alleles in one gene can be directly compared across biogeographic groups using the ‘Frequency comparison’ tool. For instance, comparing the combined frequencies of CYP2D6*3 and *4 reveals that these two loss-of-function alleles combined are most common in European and South American populations, whereas they are mostly absent or rare in East Asia, Oceania (with the exception of Australia) and Sub-Saharan Africa (Figure 3C). All plots are interactive. Hovering the cursor over the points will display a popup, showing the underlying country information. Furthermore, all underlying data can be conveniently downloaded using the ‘Download data’ button at the bottom of the page.

Variations within each geographic group can be illustrated using the ‘Intra-biogeographic variability’ tool. The display shows all populations with available frequency information for the selected allele(s) stratified by biogeographic groups. As an example, selecting CYP2D6*10 shows that this reduced function allele is most common throughout East Asia with the frequency within this population group varying between 52.4% in the Philippines and 28.8% in Indonesia (Figure 3D). While overall frequencies in North America are low (1.1–2.8%), Hawaii stands out with population frequencies of 33.3%. These results demonstrate that stratification within biogeographic groups can provide striking differences between populations and provide important information for the guidance of pharmacogenomic medicine.

The ‘Country data tool’ allows the user to select one or more genes as well as one or more countries/geographical groups to summarize all data that are available for the given selection (Figure 3E). By filtering and/or sorting the resulting table, the tool provides a convenient way to parse data across genes for given geographical selections, thus complementing the by-allele exploration on the Map&Data dashboard.

Lastly, we have implemented a ‘Metabolizer status tool’, which uses the available frequency data to infer functional consequences in one or more selected population (Figure 3F). Upon selection of a gene, the tool will display metabolizer phenotype distributions in any number of selected countries or aggregated geographical groups. The resulting data is displayed both as a stacked bar plot as well as in table format that allows for the convenient download of the results. Importantly, the functional distributions can be directly cross-referenced with the established CPIC guidelines to infer the fraction of individuals that would benefit from genetically guided selection of drugs or changes in dosing, thus providing an easily accessible overview for precision public health estimates.

### Global patterns of pharmacogenomic variability

**Figure 4.**
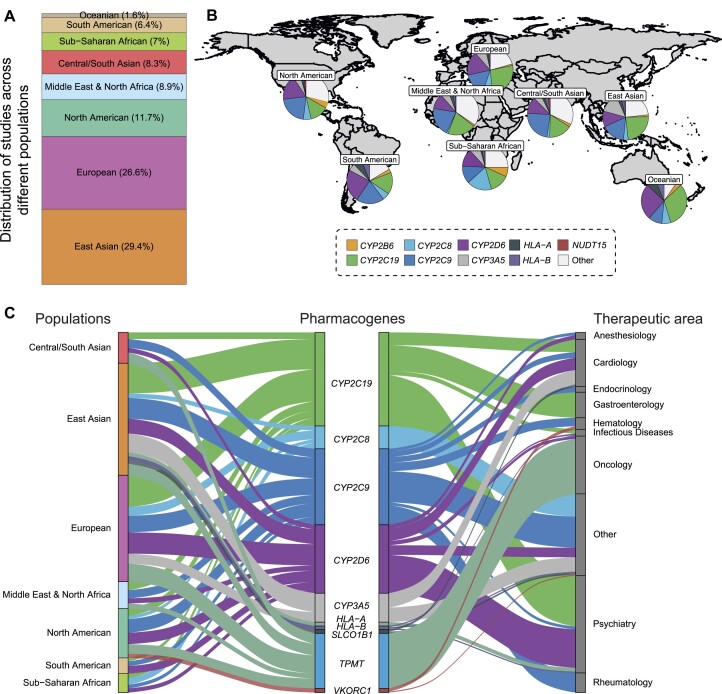
Ethnogeographic patterns of pharmacogenomic coverage. (**A**) Distribution of studies reporting pharmacogenetic allele frequencies across different biogeographic groups. (**B**) Distribution of studied pharmacogenes within biogeographic groups. Note the pronounced difference in coverage of some pharmacogenes, such as *CYP2C8, CYP2C19* and *CYP2D6*. (**C**) Sankey plot showing the landscape of pharmacogenetic coverage across populations and their relation to therapeutic areas. Note that not all genes are shown to increase visual accessibility.

When mapping the available information to the respective ethnogeographic groups, we find that most studies evaluated frequencies in East Asian and European populations, accounting for 29.4 and 26.6% of all studies, respectively (Figure 4A). In contrast, South American, Sub-Saharan African and Oceanian populations each accounted for ≤7% of all available studies. Notably, there were pronounced geographical differences in the analyzed genes (Figure 4B,C). For instance, *CYP2B6* variability is extensively analyzed in Sub-Saharan Africa, whereas this gene receives considerably lower attention in other geographical regions. Since CYP2B6 is a major determinant of the clinical metabolism of the antiretroviral efavirenz, this focus likely reflects the increased prevalence of HIV/AIDS in Sub-Saharan Africa.

**Figure 5.**
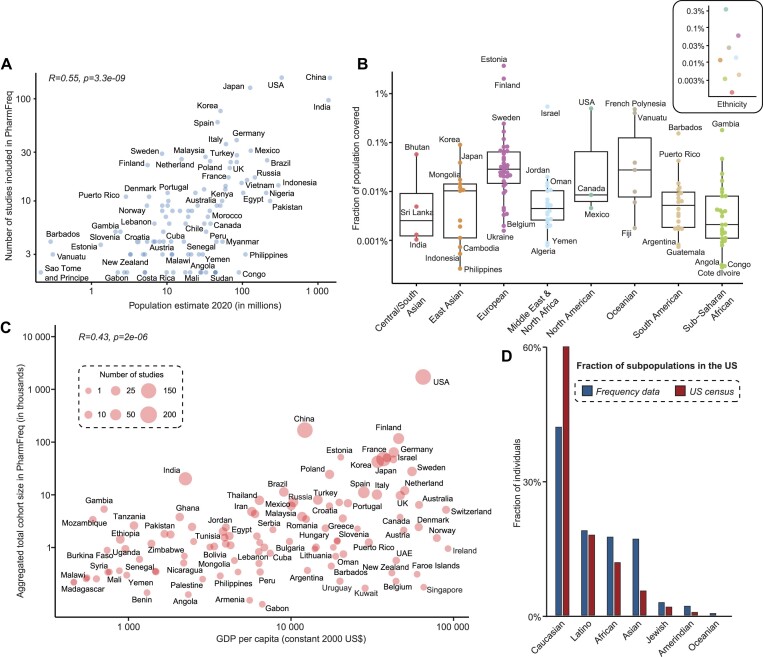
Factors impacting pharmacogenetic coverage across countries and populations. (**A**) The number of studies for each country are correlated with national population numbers (r_Spearman_= 0.55, *P*= 3.3*10^−9^). (**B**) The fraction of the general population in different countries and biogeographic groups (inlet) that are covered by pharmacogenetic studies differs widely. While average coverage for which pharmacogenetic data are published is <0.1%, for some countries, such as Finland, Estonia, Israel, USA and Vanuatu, information is available for >3% of the national population. Note that this only covers published data and does not include unpublished information, e.g. from direct to-consumer tests. (**C**) Aggregated cohort sizes correlate with the national GDP (r_Spearman_= 0.43, *P*= 2*10^−6^). The number of studies for each country were indicated by the size of the circles. (**D**) The coverage of major ethnogeographic groups in pharmacogenomic studies from the US compared to US census data. Note that available genomic information corresponds overall well to the ethnic distribution of the generation population.

The total number of studies per country mapped in the PharmFreq database correlated significantly with population size (r_Spearman_= 0.55, *P*= 3.3*10^−9^, Figure 5A). Pharmacogene frequencies in China, US, Japan, India and Korea were most extensively mapped, whereas the number of studies in Egypt, Pakistan and Nigeria were up to 10-fold lower, despite similar population sizes. Overall, the approximate population coverage was highest in Estonia, Finland, USA and Israel with available pharmacogenetic data corresponding to >0.5% of the national population size (Figure 5B). In contrast, population coverage was substantially lower in multiple African and Southeast Asian countries, such as Congo, Angola, Cote d’Ivoire, Philippines and Indonesia (data available for <0.001% of the national population). Interestingly, a similar lack of available data was also observed for some high-income countries with a GDP > 80 000 USD per capita, including Norway, Switzerland and Ireland (Figure 5C). The cohort size of admixed populations can also be compared with census data to reflect the overall representation of the different subpopulations (Figure 5D). Combined, these results demonstrate that a consolidated atlas of studies into the pharmacogenetic variability of ethnogeographic groups can reveal important inequalities, thereby facilitating focused research into underrepresented populations.

### Implementation of population-specific frequency data

#### Metabolizer phenotype estimation to inform treatment optimization

Individuals carrying alleles in drug metabolizing enzymes which results in increased or decreased activity can be at risk of adverse reactions or lack of efficacy when treated with drugs metabolized by the respective gene products. The frequencies of different reduced or increased function alleles can be aggregated to infer the prevalence of different metabolizer phenotypes at the population level. For instance, individuals carrying a functional duplication of the *CYP2D6* locus (*1xN or *2xN) are classified as ultrarapid metabolizers (UM), while individuals carrying one loss-of-function allele (e.g. *1/*5) or two decreased function alleles (e.g. *10/*10) are considered as intermediate metabolizers (IM), and patients being homozygous or compound heterozygous for loss-of-function alleles (e.g. *4/*5 or *5/*5) are defined as poor metabolizers (PM) (26). In turn, these inferred phenotypes can be translated into recommendations regarding selection and dosing of the respective drugs. For the pharmacogenes included in PharmFreq, the metabolizer status of all drug metabolizing enzymes can be calculated at the population level using the Metabolizer status tool. Thus, PharmFreq provides a valuable resource for the geographic distribution of phenotype frequencies, particularly when focusing on genes in complex loci, such as *CYP2D6* and *HLAs*, for which function is commonly assigned to haplotypes and not to single variants.

As an example, frequencies of different CYP2D6 phenotypes were calculated by electing CYP2D6 and all countries with available information (Table 3). Notably, these calculations require that frequencies for multiple functionally relevant alleles are available for a given country and, thus, metabolizer phenotypes cannot be calculated for all countries with frequency information in PharmFreq. The results show that the fraction of CYP2D6 PMs is highest in Europe and the Americas, whereas the highest fraction of UMs was found in Ethiopia and Papua New Guinea. This result is consistent with previous meta-analyses (8) but extends the number of individuals and included countries.

**Table 3.** Global distribution of inferred CYP2D6 metabolizer phenotypes. UM = ultrarapid metabolizer; NM = normal metabolizer; IM = intermediate metabolizer; PM = poor metabolizer

Population	UM	NM	IM	PM
***Europe***				
Albania	0.7%	47.3%	43%	9%
Austria	2.6%	64.1%	30.5%	2.8%
Belgium	0%	54.5%	38.9%	6.5%
Croatia	0%	61%	34.5%	4.5%
Denmark	1.1%	48.5%	42.4%	8%
Estonia	2.4%	59.8%	33.4%	4.4%
Faroe Islands	0%	41.6%	45.8%	12.6%
Finland	7.4%	61.3%	28.1%	3.2%
France	0%	52.4%	40.1%	7.6%
Germany	2.6%	51.8%	39%	6.6%
Hungary	3.2%	55.4%	36%	5.4%
Italy	2.7%	56.7%	35.8%	4.8%
Netherlands	0%	52.5%	40.6%	7%
North Macedonia	3.6%	49.8%	39.8%	6.8%
Portugal	5.2%	55.4%	34.6%	4.8%
Romania	0.8%	50.7%	43%	5.6%
Russia	3.5%	57.9%	34.1%	4.6%
Spain	5.2%	55.1%	34.8%	4.9%
Sweden	2.8%	52%	38.5%	6.8%
Switzerland	3%	49.5%	40.5%	7%
Turkey	9%	58.1%	30.4%	2.5%
***North America***				
Canada	0%	87.1%	12.5%	0.4%
Cuba	5.4%	62.7%	28.9%	3%
Hawaii	0%	33.4%	55.5%	11.1%
Mexico	3.8%	74.9%	20%	1.3%
USA	2.3%	54.5%	37.7%	5.5%
***South America***				
Argentina	0%	52.2%	40.3%	7.5%
Barbados	2.6%	57.3%	37.1%	3.1%
Brazil	2.7%	64.9%	29.8%	2.7%
Chile	0%	72.3%	25.5%	2.2%
Colombia	0%	61.8%	33.7%	4.6%
Costa Rica	5.8%	56.8%	32.7%	4.7%
Ecuador	3.9%	68.3%	25.5%	2.3%
Netherlands Antilles	0%	58.7%	37.8%	3.5%
Nicaragua	0%	63.2%	32.7%	4.1%
Trinidad and Tobago	4.7%	64%	29.3%	2%
Venezuela	0%	75.5%	22.8%	1.7%
***East Asia***				
China	0.8%	56.9%	41.7%	0.7%
Japan	1.5%	68%	30%	0.5%
Korea	1.7%	58.9%	38.9%	0.5%
***South Asian***				
India	3.8%	68.2%	26.3%	1.7%
Malaysia	0%	68.5%	31.2%	0.3%
Myanmar	0%	74.1%	25.6%	0.3%
Philippines	0%	64.8%	35.2%	0.1%
Thailand	0%	53.6%	45.7%	0.7%
Vietnam	0%	51.1%	47.8%	1.1%
***Middle East & North Africa***				
Iran	0%	73.8%	24.5%	1.7%
Iraq	0%	81.8%	17.8%	0.5%
Israel	0%	75.6%	23.9%	0.5%
Saudi Arabia	0%	85.3%	14.5%	0.2%
Syria	13.3%	67.3%	18.2%	1.2%
United Arab Emirates	10.4%	68.2%	20.6%	0.8%
***Sub-Saharan Africa***				
Ethiopia	27%	44.2%	28.2%	0.6%
Ghana	0%	66.2%	32.1%	1.7%
Kenya	0%	73.6%	26%	0.4%
South Africa	5.3%	37.1%	54%	3.6%
Tanzania	0%	67.7%	31.7%	0.6%
Zimbabwe	0%	76.8%	22.8%	0.4%
***Oceania***				
Australia	2.8%	56.4%	36%	4.9%
French Polynesia	0%	96.4%	3.6%	<0.1%
Papua New Guinea	20.9%	67.4%	11.2%	0.5%

When treated with opioids, such as codeine and tramadol, CYP2D6 UMs generate increased amounts of active metabolites, leading to a substantially increased risk of severe opioid toxidrome with respiratory depression even at labeled dosage regimens (27). In contrast, PMs generate significantly lower amounts of active metabolites and thus exhibit diminished analgesia. The aggregated PharmFreq data show that PM frequencies were highest in the Faroe Islands (12.6%), Hawaii (11.1% PMs), Albania (9% PMs) and Argentina (7.5% PMs), suggesting that these populations are more likely to experience diminished analgesia when using codeine or tramadol, whereas the risk of opioid-induced toxicity is overall highest in Ethiopia (27% UMs) and Papua New Guinea (20.9% UMs) affecting up to one in four individuals in the general population. Both PMs and UMs are strongly recommended to avoid codeine and tramadol and to rather use non-opioid analgesics or other opioids not impacted by CYP2D6 phenotype. The information in PharmFreq can thus be used to accurately estimate genetically encoded risk with high ethnogeographic resolution at the population scale.

#### Guiding population-specific genotyping strategy

The optimization of pharmacogenetically guided treatment strategies is highly dependent on the prevalence of the respective risk alleles in the population of interest. The PREPARE study was the largest pharmacogenetic implementation trial in Europe and evaluated the benefits of pharmacogenetic genotyping of a panel of 50 variants across seven European countries (28). While it is clear that frequency is an important factor for determining which alleles should be included in such panels, the information should be derived from the countries in which the tests are to be implemented (29). By exploring the frequency data in PharmFreq, regional and population differences can be easily identified. For example, while the increased function allele CYP2C19*17, which is included in the pharmacogenetic guidelines of 14 drugs, is highly prevalent in European, African and American populations with frequencies pivoting around 17–33%, its frequency is substantially lower in Japan (1.1%), China (1.2%) and Southeast Asia (1–4.7%). Similarly, frequencies of the *DPYD* HapB3 genotype, which is important to consider for the therapy with 5-fluorouracil and related drugs, are relatively high across Europe (1–3.8%), whereas the allele is virtually absent across East Asia. In contrast, the frequency of the decreased function allele CYP2D6*10 is very high (29–52%) in East Asians, but substantially less common in Central and South Asia (3–13%), Europe (1–3%) and the Americas (1–7%). In addition to the exploration in the PharmFreq Map interface, the Country Data tool provides an easy way to identify the most frequent pharmacogenetic variations in one or more countries of interest. For instance, a selection of *CYP* genes in China reveals that CYP2D6*10 constitutes the most common functionally important pharmacoallele in this population (47.1%), followed by CYP2C19*2 (29.3%) and CYP2B6*6 (16.3%). Such information can thus provide powerful information for the composition of population-specific genotyping panels to more efficiently capture pharmacogenetic phenotypes in the country or regions of interest.

#### Cost-effectiveness analysis for preemptive genotyping

One of the major considerations for the clinical implementation of pharmacogenomics is whether the respective tests are cost-effective. This is typically evaluated by comparing the costs of the current prescribing practice to the cost of a genetically guided strategy. Importantly, the frequency of tested alleles in the studied population has major influence on the cost structure of the genetic strategy. This can be intuitively explained as genetic testing of a very rare allele, requires many more tests to be conducted (and thus much higher costs) to identify a risk allele carrier who might benefit from genetically guided dosing adjustments. For instance, PharmFreq reveals high frequencies of HLA-B*15:02, the risk allele for severe carbamazepine intolerance, in Burma (10.8%), Thailand (8.3%), Vietnam (13.5%), Indonesia (11.5%) and the Philippines (22%), whereas frequencies in India (1.9%), China (3.8%), Japan (<0.1%) and other geographical groups (all <1%) are substantially lower. Factoring in such data into established cost-effectiveness models for carbamazepine therapy reveals that preemptive genotyping of HLA-B*15:02 is only cost-effective in Southeast Asia but not in other populations, as reported previously (30). By serving as a living resource for the global distribution of pharmacogenetic allele frequencies, PharmFreq can provide critical input for the refinement of cost-effectiveness models not only for HLA alleles but also other treatment-relevant pharmacogenes.

## Conclusions and future perspectives

Sequencing resources and genotype–phenotype catalogs were long based on study populations that were almost exclusively of European descent. However, with increasing throughput of genomic profiling methods awareness increased that this biased perspective is inadequate for accurate understanding and predicting human phenotypes, disease risk and drug response (31–33). While a multitude of meta-analyses have aimed to consolidate the available literature and to map the variability in clinically important pharmacogenes, these were limited to individual genes (8,34–36) or geographic regions (37–39). PharmFreq builds on this available wealth of data by providing a consolidated, one-stop, free and openly accessible repository for pharmacogenomic country-specific allele frequencies. These aggregated data allow to identify and quantify important research gaps and thus can facilitate the appropriate allocation of future research resources to provide equitable pharmacogenomic benefits.

## Supplementary Material

gkae1016_Supplemental_Files

## Data Availability

PharmFreq is accessible without need for registration and free of charge at pharmfreq.com. We are fully committed to Open Science and, as such, all relevant codes and underlying resources are made publicly available on Github at https://github.com/ikp-stuttgart/pharmfreq and Zenodo at https://doi.org/10.5281/zenodo.13935076.
