# Supplementary material for: PharmFreq: a comprehensive atlas of ethnogeographic allelic variation in clinically important pharmacogenes
Source: Nucleic Acids Res. 2024 Nov 14;53(D1):D1498–509. doi: 10.1093/nar/gkae1016 (PMC11701539; doi:10.1093/nar/gkae1016)
Supplement: gkae1016_Supplemental_Files [file gkae1016_supplemental_files.zip › Supplementary Table 1 legends.docx]

**Supplementary Table 1: List of all studies included in PharmFreq.**
